# Optimal viewing angles of intraoperative fluoroscopy for detecting screw penetration in proximal humeral fractures: a cadaveric study

**DOI:** 10.1186/s12891-018-2247-8

**Published:** 2018-09-07

**Authors:** Qiuke Wang, Yifei Liu, Ming Zhang, Yu Zhu, Lei Wang, Yunfeng Chen

**Affiliations:** Department of Orthopedic Surgery, Shanghai Jiao Tong University Affiliated Sixth People’s Hospital, 600 Yishan Road, Shanghai, 200233 People’s Republic of China

**Keywords:** Proximal humeral fracture, Screw penetration, Complication, Fluoroscopy, Surgery, Trauma

## Abstract

**Background:**

To identify the optimal viewing angles for every proximal screw in PHILOS plate-fixed proximal humeral fractures.

**Methods:**

Three fresh-frozen human cadaveric bodies with six intact shoulders were studied. All three bodies were put in the beach chair position and PHILOS plates were placed on the proximal humerus. Head screws penetrating 1 mm into the joint were fitted one by one. Fluoroscopy was conducted in the 180° horizontal plane and the 120° coronal plane to analyze each screw’s penetration in every shoulder. Images were taken every 5°, then all images were analyzed to identify the sensitive angles.

**Results:**

The range of optimal viewing angles to visualize penetration of every head screw was identified. In the coronal plane, the angles in the range between 0° and 10° were sensitive to all screws except No. 8 and No. 9. Furthermore, penetration of screws No. 8 and 9 could not be identified on any axillary view, but could be identified in the horizontal plane from − 30° to − 10° and from 10° to 35° respectively.

**Conclusions:**

We recommend a 0°–10° axillary view with 30° arm abduction combined with two horizontal angles in the range of − 30° to − 10° and 10° to 35° for routine fluoroscopy during surgery. Our results will be helpful in avoiding primary screw penetration.

## Background

Proximal humeral fracture accounts for approximately 5% of all bone fractures, and the rate is rising with the increasing age of the population [1–3]. Although roughly 80% of these fractures can be treated conservatively, the rest, which are considered as displaced fractures, are recommended to be fixed by surgery [4]. Since locking plates, especially the PHILOS plate (Proximal Humerus Internal Locking System, Depuy Synthes, Warsaw, IN, USA), are becoming more and more popular to achieve satisfactory outcomes in fixing proximal humeral fractures [5–8], a high rate of complications has been reported related to the use of plates, of which screw penetration is the most frequent [4, 6, 9–11]. Patients with screw penetration may experience severe pain and require subsequent revision surgery to remove the internal plate, which has a significant effect on rehabilitation. Screw penetration includes both primary and secondary screw penetration. The incidence of primary screw penetration is proximately 14% [5] it occurs during surgery and is considered to be an avoidable complication, preventable by careful operation and appropriate fluoroscopic detection during surgery. However, knowledge of the appropriate fluoroscopic detection is inadequate. Spross et al. [12] found that only the combination of four projections of fluoroscopic detection (neutral, 30° external rotation, 30° internal rotation and axial in 30°) could achieve 100% sensitivity and that standard radiographs (anteroposterior and outlet) missed almost half the instances of primary screw penetration in fractures fixed with the PHILOS plate. The process required to complete four angles of fluoroscopic detection during surgery is tedious and time-consuming, which means that primary screw penetration can easily be missed during routine surgeries. Two studies of Theopold et al. reported that intraoperative 3D fluoroscopy enabled 100% detection of the screw perforations [13, 14]. However, 3D fluoroscopy equipment was not available in most operating rooms while regular X-ray projector (c-arm) was always equipped.

The aim of our study therefore was to explore the optimal viewing angles of intraoperative fluoroscopy for every proximal screw in PHILOS plate-fixed proximal humeral fractures, and then suggesting some intra-operative fluoroscopy angles for avoiding screw penetration.

## Methods

This study was approved by ethics of committee of Shanghai sixth people’s hospital, the approval number is 2016-ky-005, and all donors had signed agreement about further cadaveric research and education before body donation. Three fresh-frozen human cadaveric bodies with six intact shoulders were obtained, which comprised 2 females and 1 male, with a mean age of 72.2 years (range 64–82). All the specimens included the entire scapula, clavicle, humerus and intact associated soft tissues. Gross examination was performed and clinical histories were reviewed to exclude any history of bone diseases.

### Preparation of specimens

All three bodies were put in a beach chair position to simulate the intraoperative situation. The PHILOS plates were placed on the proximal humerus, 5 mm lateral to the bicipital groove and 8 mm distal to the tip of the greater tuberosity, in all shoulders through the standard deltopectoral approach. Two non-locking screws were initially placed in the shaft holes to fix the plates in position. Second, we simulated the methods used in the study by Spross et al. [12]; the subscapularis muscle was tenotomized close to the lesser tuberosity and the anterior capsule was vertically incised to gain full sight into the joint and of the humeral head, so that drilling of head screws could be visually controlled to avoid perforating the head. Lastly, the lengths of the head screws were chosen according to the measurement of the lengths of the drill holes, and nine head screws were placed in the humeral head in each shoulder.

### Imaging investigation

**Fig. 1:**
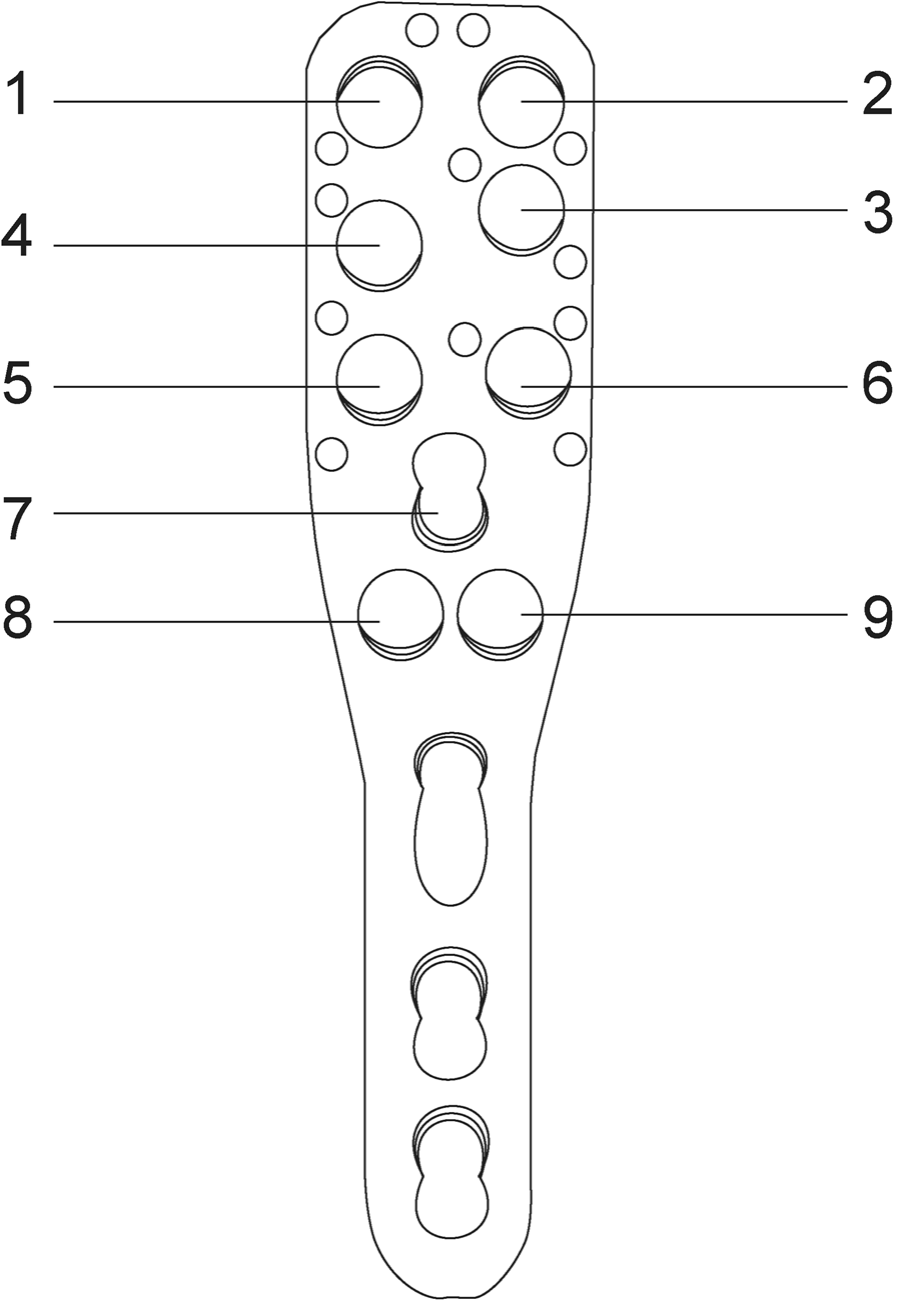
The numbering of the head screws (holes) on the PHILOS plate

**Fig. 2:**
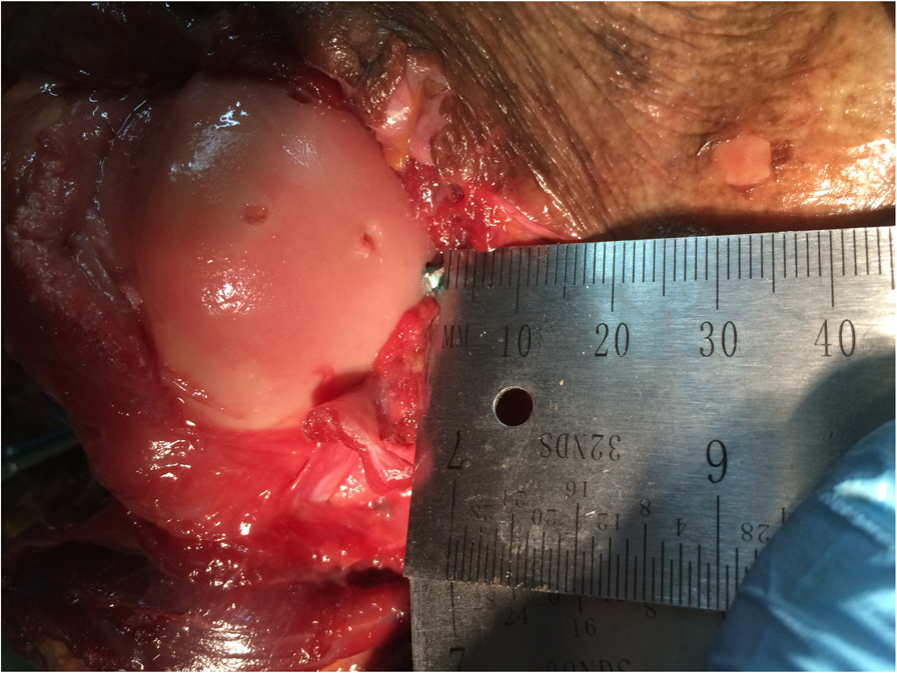
Each head screw penetrates the humeral head to 1 mm under visual control

**Fig. 3:**
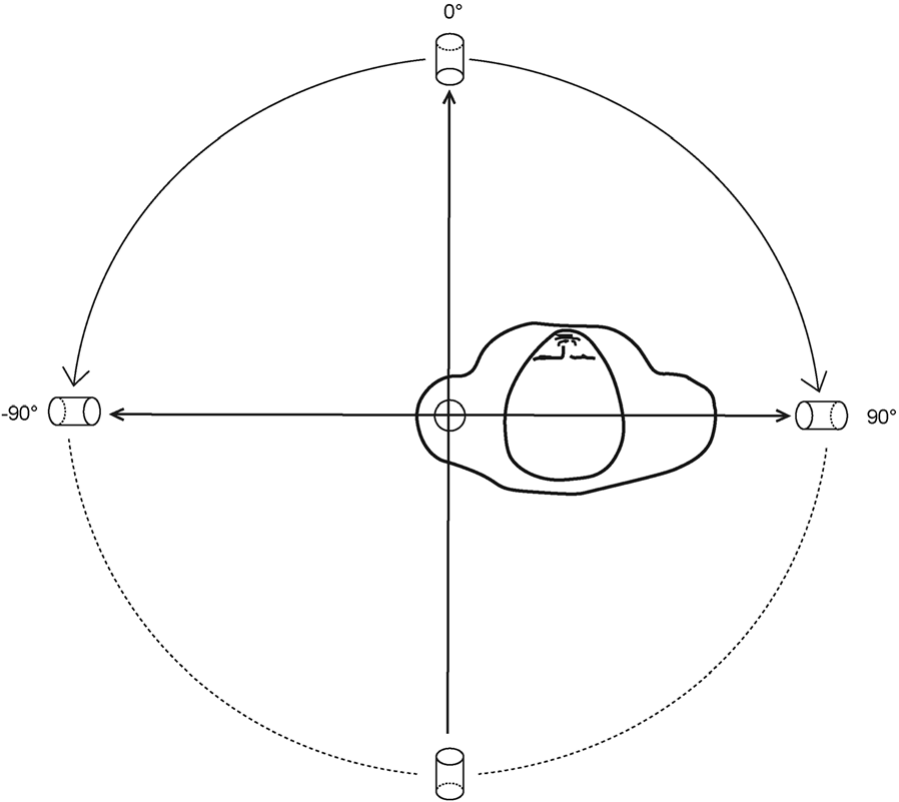
Taking the left shoulder as an example, radiographic projection was conducted in a 180° horizontal plane. Radiographic images were taken every 5°

Head screws were numbered to facilitate identification and recording (Fig. 1). The No. 1 screw was removed (prepared above) and a longer screw was inserted to penetrate the head, the length was controlled to penetrate 1 mm beyond the surface of the head under direct vision (Fig. 2). After exchanging the screws, the joint was sutured and the arm was placed in a neutral position. To simulate the anteroposterior projections, the c-arm was positioned in the anteroposterior direction (perpendicular to coronal plane of the body and c-arm on the horizontal plane) and it was set as 0°. Then the c-arm was rotated clockwise (overlooked), and an image was obtained every 5° up to 90°, resulting in 18 images being obtained. After that, the c-arm was reset at 0° and rotated anti-clockwise up to 90°, and again an image was obtained every 5°. In total, 37 images were obtained in the 180° horizontal plane. In order to facilitate data recording, for the left shoulder we set the angle on the right as “+” and on the left as “-”; we then used the opposite convention for the right shoulder (Fig. 3). During this process, the body and the arm were kept still.

**Fig. 4:**
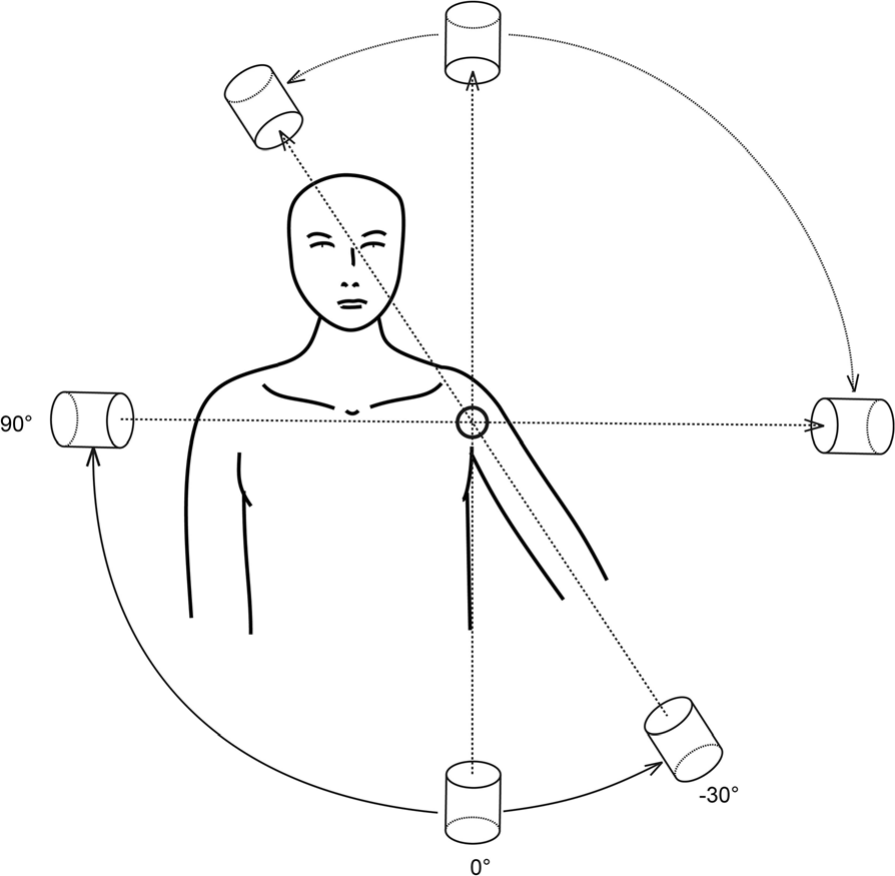
Taking the left shoulder as an example, the arm was positioned at 30° of abduction, and radiographic projection was conducted in a 120° coronal plane. Radiographic images were taken every 5°

In order to simulate the axillary view, the c-arm was placed on the coronal plane of the body and the arm was placed at 30° of abduction (Fig. 4). Taking the left shoulder as an example, the standard axillary view was set as 0°, then the c-arm was rotated clockwise (sighted from the anterior to the posterior of the body), and an image was obtained every 5° up to 90° (equivalent to the axillary view with the arm at 120° of abduction). After that, the c-arm was reset at 0° and rotated anti-clockwise up to 30° (equivalent to the axillary view with the arm at 0° abduction) and an image was obtained every 5°. A total of 25 images were obtained on the 120° coronal plane, and as above, in the left shoulder we set the angle on the right as “+” and on the left as “-”, while for the right shoulder, the opposite convention was used. During this process, the body was kept still with the arm in 30° of abduction.

Overall, a total of 62 images were obtained for the penetration of No. 1 screw. The same method was then applied for the imaging investigation of penetration of the next 8 screws, to give a total of 558 images from one shoulder. All 6 shoulders underwent the same process and 558 × 6 images were collected.

### Imaging analysis

All images were numbered and were independently and randomly reviewed for screw penetration (yes/no) by two experienced attending doctors. The two doctors were blinded to the experimental design and process. All discordant cases were reevaluated by a third examiner, and the results were assigned by consensus of the three examiners. Based on these results, for each shoulder, we were able to draw a viewing angle range to detect penetration of every head screw.

### Statistical analysis

**Fig. 5:**
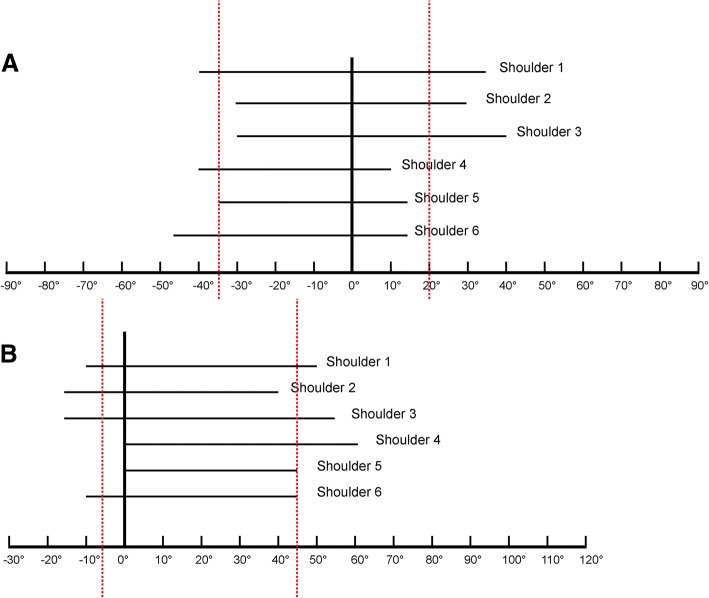
For each shoulder, the range of sensitive angles of the No. 1 screw is presented by a solid black line, and the two dotted red lines present the average minimum and maximum sensitive angles of the 6 shoulders. **a** Horizontal plane; **b** Coronal plane

Statistical analysis was performed using the statistical program SPSS version 20.0 (IBM Corp., Armonk, NY, USA). The optimal viewing angle range of every screw was set between the average minimum and maximum of the six shoulders. In this study each screw has an angle range in each shoulder, so the average of the six angle ranges was calculated for each screw, with the lower boundary set as the average of the lower boundary of the six angle ranges and the higher boundary as the average of the higher boundary of the six angle ranges. In order to simplify clinical application, all the angles were measured to the nearest multiple of five greater than the average lower value for the lower boundary, for example if the average value was 8 we chose 10; and we chose the first multiple of five less than the average higher value for the higher boundary, for example if the average value was 8 we chose 5 (Fig. 5).

## Results

**Fig. 6:**
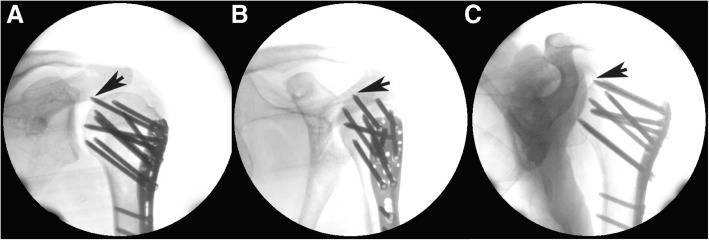
Three images present three different viewing angles in the horizontal plane for the No. 1 screw in shoulder 1. Screw penetrations can be identified in three viewing angles, **a** 0°; **b** 40°; **c**: -35°

As each screw was positioned one by one to penetrate the humeral head in each shoulder, the same radiographic process was performed as described above, and screw penetrations could be identified on the images (Fig. 6).

**Fig. 7:**
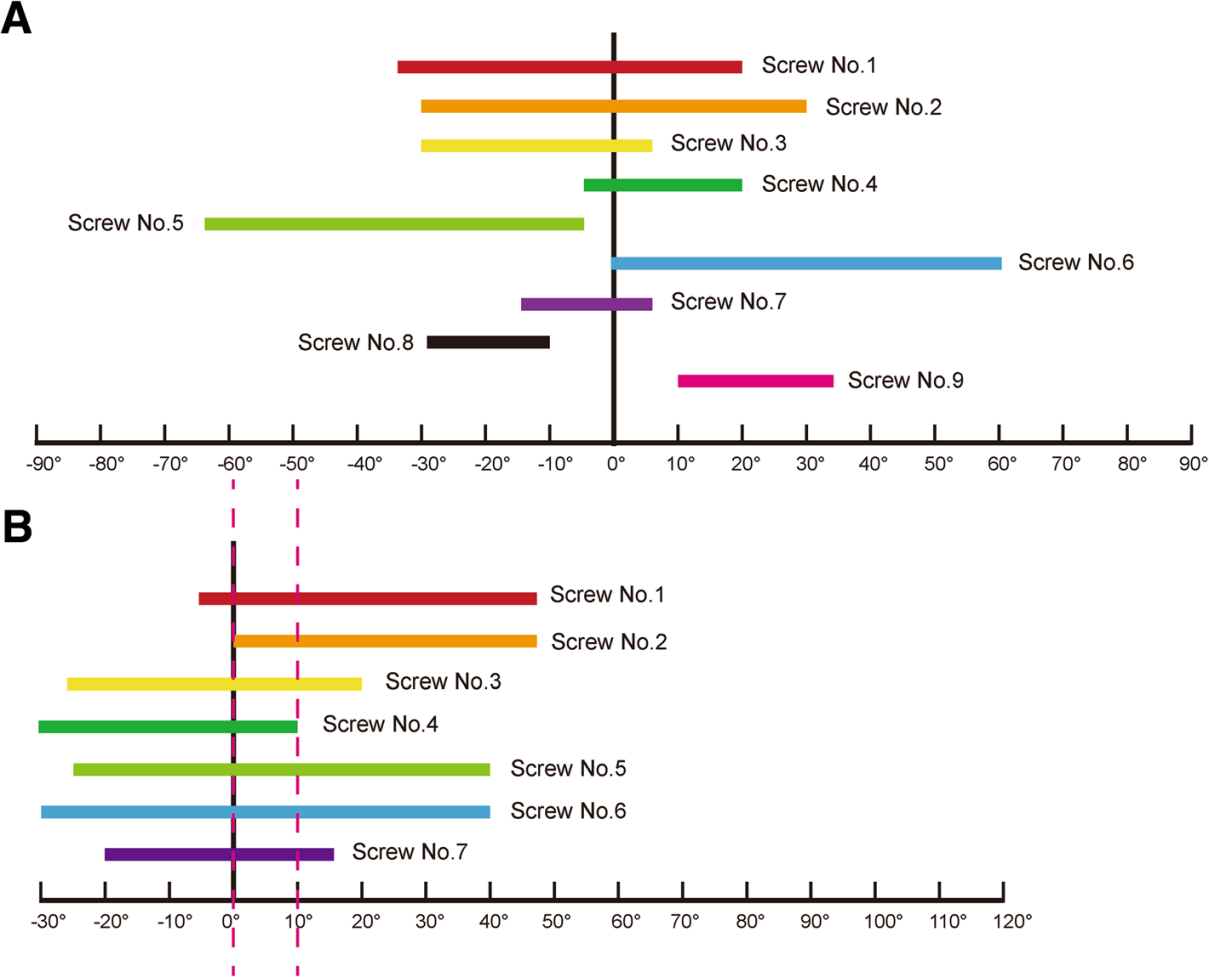
The optimal viewing angles for each screw are indicated in the diagram by colored bands. **a** Horizontal plane; **b** Coronal plane. The angles between the two dotted red lines provide sensitive visualization of all seven screws

**Fig. 8:**
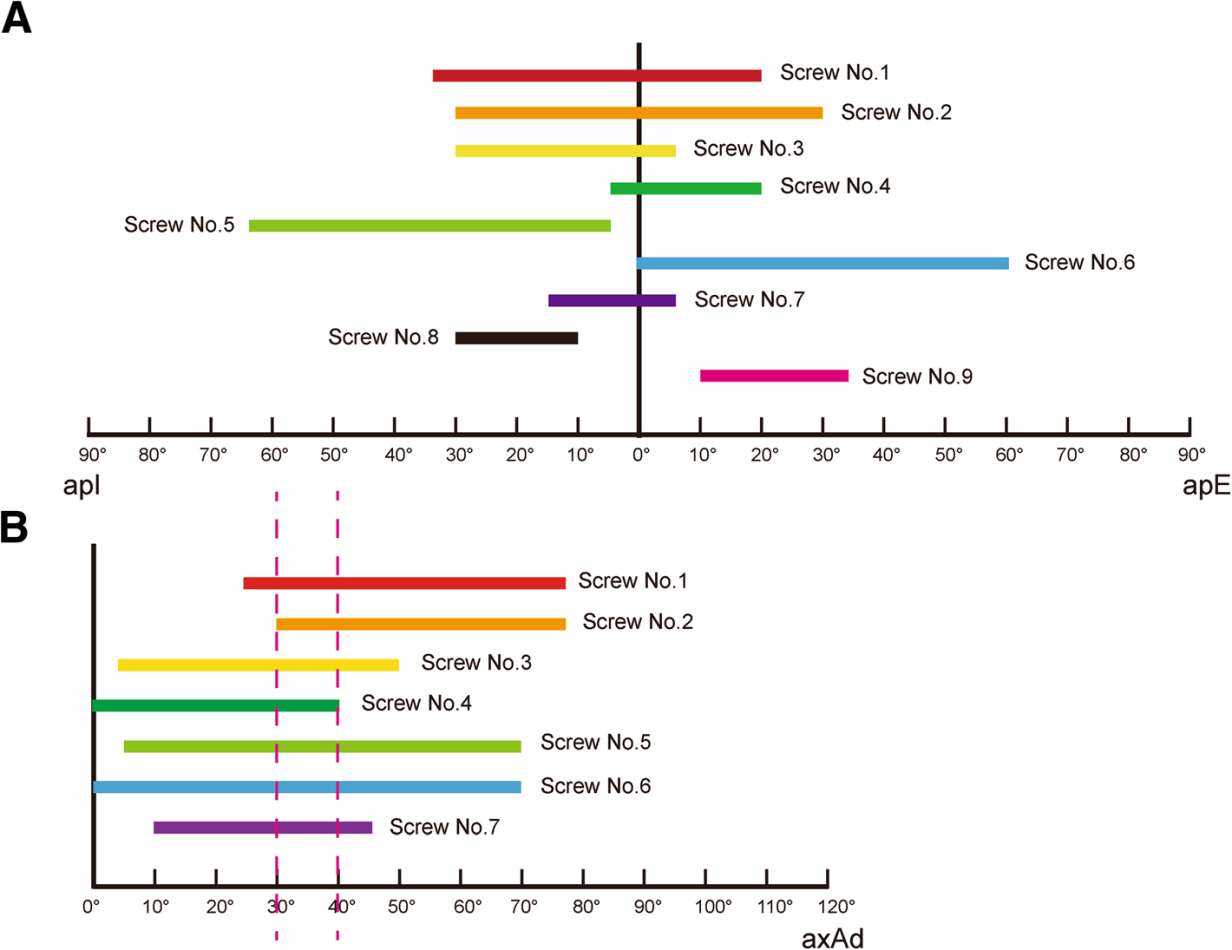
The data in Fig. 7 can be converted into another type by swinging the arm rather than rotating the c-arm. apI: internal rotation of the arm in anteroposterior projections; apE: external rotation of the arm in anteroposterior projections; axAd: external abduction of the arm in axillary projections

After calculating the average minimum and maximum sensitive angles of all six shoulders, the optimal range of viewing angles to visualize penetration of every head screw was identified. Taking No. 1 screw as an example, the viewing angles ranged from − 35° to 20° in the horizontal plane (Fig. 5a) and − 5° to 45° in the coronal plane (Fig. 5b). All viewing angle ranges are collated in Fig. 7. In the coronal plane, the angles in the range between 0° and 10° were able to visualize all screws except No. 8 and 9. Furthermore, penetration of screws No. 8 and 9 could not be identified on any axillary view, but could be identified in the horizontal plane only. In this study, we maintained the body and arm in a fixed position, therefore theoretically, our angle data could be transferred to another type of fluoroscope in which we could rotate the arm or move it without moving the c-arm. The internal and external rotation of the arm in anteroposterior projections is equal to the rotation of the c-arm in the horizontal plane, and the transferred data are presented in Fig. 8.

## Discussion

The PHILOS plate is a widely-used implant for fixing proximal humeral fractures; it is designed to position divergent screws in the humeral head in order to increase screw purchase; however, its unique design makes it difficult to identify the screws’ position during surgery. Screw penetration is found to occur frequently during surgery as well as at follow-up, resulting in damage to the glenoid fossa caused by the screw tip and causing the patient to suffer severe pain [14–18]. Screw penetration of PHILOS plate is the primary object of our study, and our results concerning the optimal viewing angle ranges for intraoperative fluoroscopy to detect screw penetration lead the way for surgeons. Traditional standard anteroposterior and lateral fluoroscopy may miss penetration of at least 3 screws (No. 5, 8 and 9), while for three orthogonal views, in the trauma-series of x-rays (true glenoid anteroposterior, trans-scapular lateral and axillary views), penetration of screw No. 8 can still be missed. This may be the reason why screw penetration remains the most frequent complication. Penetration of screws No. 8 and 9 could not be identified on our axillary views; this may be because these two screws are positioned on the lower part of the spherical head, so that the superior part may easily hide the 1 mm cut out and our view angles are limited between − 30°and 90°.

Some instances of penetration found at follow-up may have happened during surgery and have been missed because of inadequate fluoroscopy. In a study by Charalambous et al. [5], 4 of 25 patients were found to have screw penetration at follow-up, and subsequent review of the intra-operative radiographs showed that the penetration was missed intra-operatively in one case. Thanasas et al. [19] highlighted the use of intraoperative fluoroscopy and recommended fluoroscopy in at least two different planes to avoid screw penetration. However, in their study, little information is available about which angles or planes have a high sensitivity for detecting screw penetration during surgery. Spross et al. [12] conducted a study to test some widely-used radiographic projections for detecting screw penetration, and found that anteroposterior and outlet radiographs, especially in internal rotation, may miss almost half the instances of screw penetration, while the standard axillary view (30° abduction) presented the highest sensitivity in a single radiographic projection.

According to our data, the standard axillary view with the arm at 30° abduction should be recommended, and the angles in the range between 0° and 10° (abduction 30–40°) show the same high sensitivity. In the horizontal plane, at least two different projections should be used to detect screws No. 8 and 9, one in the range between − 30° and − 10°, another between 10° and 35°, which equate to internal rotation of the arm 10–30° and external rotation of 10–35°. In addition, we found that the standard lateral view was not able to identify any screw penetration, which may be because 1 mm is too short to be detected. We recommend that our results should be used intraoperatively to reduce the rate of primary screw penetration rather than at follow-up. This is because, on the one hand, a large proportion of secondary screw penetrations are secondary to severe humeral head deformities, such as varus and necrosis, so that the optimal angles for these heads would be different from our data, while on the other hand, it may be impractical to perform fluoroscopy using such accurate angles at each follow-up.

In the process of carrying out this study, we chose to rotate the c-arm rather than rotate the arm (internal and external rotation), because it is easier and more precise to control the angle according to the angle indexes on the c-arm, and it is also more practical during surgery.

Based on our results, we can conclude that the sensitivity of every radiographic projection, for anteroposterior and outlet radiographs, means that penetration of three screws may be missed, while the result of a standard axillary view is similar to that found in the study by Spross et al., showing that it is sensitive to all screws but No. 8 and 9. So we recommend 0°–10° axillary view with the arm at 30° abduction combined with two horizontal angles in the range of − 30° to − 10° and 10° to 35° as a good combination; however, this is not the only one which can achieve 100% sensitivity, because the standard 0° axillary view with the arm at 30° abduction is used in many places and is easy to remember.

There are some limitations to this research. Firstly, the protocol was designed based on healthy shoulders with no history of bone diseases, so we simulated an anatomical reduction model. Although for some fractures it is difficult to obtain the absolute anatomical reduction [20], we believe a slight deformity can be acceptable under the angle ranges. Secondly, there are wide variations in the length of the screw tip clinically cut out of the head; we set all screws cut out of the head to 1 mm, so shorter penetrations can still be missed under these views. Finally, we studied only one kind of plate. Although it is used commonly, the screws on this plate are divergently positioned in every section of the humeral head, so its unique design means that our data may be unsuitable for application to other implants.

## Conclusion

In conclusion, after studying the viewing angles of every proximal screw on PHILOS plate-fixed proximal humeral fractures, we obtained an optimal viewing angle range for every screw. Consequently, we recommend 0°–10° axillary view with the arm at 30° abduction, while another two horizontal angles in the range of − 30° to − 10° and 10° to 35° should be included. Our results will be helpful for avoiding screw penetration during surgery.

## Abbreviation

PHILOS: Proximal Humerus Internal Locking System
